# Diagnostic utility of serum ferritin in culture proven bacterial infection: an observational study

**DOI:** 10.1186/s12879-026-13298-3

**Published:** 2026-04-15

**Authors:** Pooja Gehlot, Sunil Kumar Kasundriya, Shreya Shrivastava, Manju Purohit, Shweta Khare, Ashish Pathak

**Affiliations:** 1 Department of Pediatrics, Ruxmaniben Deepchand Gardi Medical College, Ujjain, Madhya Pradesh 456006 India; 2 Department of Global Public Health, Health Systems and Policy (HSP): Medicines, focusing antibiotics, Karolinska Institutet, Stockholm, 17177 Sweden; 3 Department of Pathology, Ruxmaniben Deepchand Gardi Medical College, Ujjain, Madhya Pradesh 456006 India; 4 Department of Public Health and Environment, Ruxmaniben Deepchand Gardi Medical College, Ujjain, Madhya Pradesh 456006 India

**Keywords:** Neonatal sepsis, Sepsis screen, Serum ferritin, Youden Index, Resource-limited setting, India

## Abstract

**Introduction:**

Diagnosing neonatal sepsis remains challenging due to the low yield of blood cultures. Most neonatal intensive care units (NICUs) rely on a sepsis screen for diagnosis. This study aimed to evaluate the diagnostic utility of serum ferritin as a standalone marker and in combination with the existing septic screen.

**Methods:**

This prospective study was conducted between August 2023 and January 2024 in a level-III NICU and included 160 neonates with suspected sepsis who stayed in the NICU for more than 48 h. Sensitivity, specificity, and the area under the receiver operator curve (ROC) for serum ferritin were calculated in relation to culture-proven bacterial infection. The Youden Index was used to determine the optimal serum ferritin cut-off value.

**Results:**

The mean age of the neonates was 1.83 ± 2.26 days; 64% were boys, 69% were term, and 54% had low birth weight. Among the outcomes, 28% had positive blood cultures, 45% were septic screen positive, and 27% had clinical sepsis. The mean serum ferritin level was significantly higher in septic screen-positive neonates (426.9 ng/mL) than in culture-positive cases (326.61 ng/mL; *p* < 0.001). In neonates with blood culture-positive sepsis, a serum ferritin cut-off value of > 295.5 ng/mL yielded an Area Under Curve (AUC) of 0.70, sensitivity of 54%, and specificity of 86% (Youden Index: 0.403). The sepsis screen alone had an AUC of 0.58, sensitivity of 57%, and specificity of 60%. When serum ferritin was added to the septic screen, the AUC remained similar (0.59), sensitivity increased to 72%, but specificity decreased to 46%.

**Conclusion:**

Serum ferritin levels were higher in septic screen-positive sepsis than in culture-positive sepsis. Adding serum ferritin (cut-off: >295 ng/mL) to the septic screen improved its sensitivity, albeit with reduced specificity. Serum ferritin has limited role adjunctive value, can have a role as a “rule - out” marker in diagnosing culture positive bacterial infection.

**Supplementary Information:**

The online version contains supplementary material available at 10.1186/s12879-026-13298-3.

## Introduction

Sepsis is a dysregulated host response to infections leading to life-threatening organ dysfunction [1]. Neonatal sepsis remains a major cause of morbidity and mortality during the neonatal period, particularly in low- and middle-income countries (LMICs) [2]. The burden of infection is especially high in Southeast Asia, with disability-adjusted life years (DALYs) reported at 180,599.79 per 100,000 [2]. While the incidence of neonatal sepsis in high-income countries ranges from 1 to 4 cases per 1,000 live births, it can reach 49–170 cases per 1,000 live births in LMICs, with case fatality rates up to 24% [3]. India has the highest incidence of clinical neonatal sepsis, estimated at 17,000 per 100,000 live births, with case fatality rates ranging from 25% to 65% [4].

Neonatal sepsis refers to a clinical condition related to bacteremia occurring within the first 28 days of life, typically presenting with systemic signs of infection [5]. It may involve septicemia, meningitis, pneumonia, arthritis, osteomyelitis, and urinary tract infections [5].

The clinical signs and symptoms of neonatal infections are often vague and nonspecific, including hypothermia or hyperthermia, lethargy, weak cry, poor feeding, poor perfusion, prolonged capillary refill time (> 3 s), hypotonia, absent neonatal reflexes, bradycardia or tachycardia, respiratory distress, apnea or gasping respiration, hypoglycemia or hyperglycemia, and metabolic acidosis, rendering diagnosis in the early stage difficult [3, 5–7].

Although blood culture remains the gold standard for diagnosing neonatal sepsis [3], its sensitivity is often limited due to factors such as insufficient blood volume, low colony counts, transient bacteremia, prior antibiotic exposure, and inadequate laboratory facilities especially in LMICs [3]. Clinically diagnosed neonatal sepsis can affect up to 170 per 1,000 live births, while blood culture-confirmed sepsis is as low as 5.5 per 1000 live births [8]. Consequently, most Indian NICUs depend on a composite “sepsis screen” comprising total leukocyte count, absolute neutrophil count, immature-to-mature neutrophil ratio, micro-erythrocyte sedimentation rate (ESR), and C-reactive protein (CRP) [5]. However, this screen has limited sensitivity and specificity for the diagnosis of neonatal sepsis. Overreliance on it has led to a substantial increase in empirical antibiotic use—up to 10-fold—which can disrupt gut microbiota and contribute to increased mortality [9, 10], poor neurodevelopmental outcomes [11], and the emergence of multi-drug-resistant organisms [8]. Improving the sensitivity and specificity of the septic screen is thus urgently needed [3, 6, 8, 12].

The sepsis screen in this study, based on the National Neonatology Forum (NNF) guidelines [5], comprises total leukocyte count, absolute neutrophil count, immature-to-mature neutrophil ratio, micro-erythrocyte sedimentation rate (micro-ESR), and C-reactive protein (CRP). Procalcitonin was not included due to its limited availability in our resource-limited setting and its reported variable sensitivity in early-onset neonatal sepsis, which may reduce its utility in our context [3, 6, 12].

Serum ferritin, an acute-phase protein, is elevated in various inflammatory and infectious diseases, including systemic lupus erythematosus, rheumatoid arthritis, cancers, cardiovascular conditions, viral infections (e.g., COVID-19), tuberculosis, malaria, and sepsis [13]. In critically ill patients, elevated ferritin levels correlate with disease severity, making it a promising diagnostic and prognostic marker [14]. Despite this, ferritin’s role in neonatal sepsis remains underexplored [3]. This study aimed to evaluate the diagnostic utility of serum ferritin in culture-confirmed neonatal sepsis and to assess the effect of incorporating ferritin into the existing septic screen.

## Materials and methods

### Study site and duration

This hospital-based prospective study was conducted in the 18-bedded level III Neonatal Intensive Care Unit (NICU) of C.R. Gardi Hospital, a teaching hospital affiliated with R.D. Gardi Medical College, Ujjain, India. The study was carried out over a 6-month period from August 2023 to January 2024.

### NICU admission and sepsis evaluation protocol

Neonates were admitted to the NICU based on standard institutional criteria, including prematurity, low birth weight, respiratory distress, birth asphyxia, hemodynamic instability, or clinical suspicion of infection. All neonates with suspected infection underwent a standardized clinical assessment at admission or at the onset of sepsis-related symptoms.

Blood culture, complete blood count, C-reactive protein, septic screen parameters, and serum ferritin were obtained at the time of initial sepsis evaluation, which was done within 24 h of admission, prior to initiation of antibiotic therapy. Repeat investigations were performed at 24–72 h based on clinical judgment and response to treatment, in accordance with unit protocol.

### Study population and data collection

All consecutive neonates with suspected sepsis (regardless of gestational age) who remained in the NICU for more than 48 h were included. Exclusion criteria included transfer from another NICU, presence of lethal congenital anomalies, extremely low birth weight neonates, or parental refusal to provide consent.

After obtaining written informed consent, a structured questionnaire was completed for each participant (Supplementary File 1). Data collected included demographic details, maternal history, associated perinatal risk factors, and clinical features. Birth weight was measured using an electronic weighing scale, and gestational age was assessed using the Modified Ballard Score. Neonates were classified using Lubchenco growth charts.

### Laboratory investigations

The following investigations were performed for all neonates: (a) Complete Blood Count (CBC) done using a five-part automated coulter counter (XS-800i, Sysmex India Pvt. Ltd., India) to assess total leukocyte count, differential count, and platelet count; (b) Peripheral Smear Examination: Blood smears were stained and examined microscopically for leukocyte morphology, immature-to-mature cell ratio, and toxic granules; (c) Quantitative C - reactive protein (CRP): measured using Vitros CRP slides on the Vitros 3600 Chemistry Analyzer (Ortho Clinical Diagnostics, Johnson & Johnson, USA); (d) Serum ferritin: quantified using the Vitros3600 Chemistry Analyzer (Ortho Clinical Diagnostics, Johnson & Johnson, USA); (e) Blood culture: samples were collected in BactecPeds Plus/F vials^®^ and processed using the automated BacT/ALERT system (bioMérieux, Inc., Marcy l’Étoile, France) to isolate bacterial and fungal pathogens. Blood samples and cultures were obtained upon admission or at the onset of sepsis (at 24 and 72 h). Facilities for viral cultures were not available; (f) additional investigations such as random blood sugar (RBS), chest radiography, transcranial ultrasonography (USG), and other clinically indicated procedures were conducted based on the presenting symptoms. About 2 ml of blood was collected for hematological and biochemical tests and 3–4 ml for blood culture. All samples were collected under sterile conditions.

### Timing of sepsis screen

The sepsis screen was sent after the clinician suspected sepsis and before any blood product were given to neonates.

### Operational definitions

#### Case definition

Infants less than 28 days of either sex with clinically suspected sepsis born in C.R. Gardi Hospital associated with R.D. Gardi Medical College, Ujjain.

#### Neonatal sepsis

Defined as the presence of bacteria (culture positive) in sterile body fluids like blood, urine, CSF, pleural fluid, or peritoneal fluid [5]. Neonatal sepsis includes septicemia, pneumonia, meningitis, osteomyelitis, arthritis, and urinary tract infection [5].

#### Clinical sepsis

Clinical sepsis was defined according to NNF guidelines [5] as the presence of clinical signs or symptoms with no other recognized cause: tachycardia (heart rate > 160/min), tachypnea (RR > 60/min) and temperature instability (fever > 100.4 F) or hypothermia (< 36.5–97.7 F).

##### Birth asphyxia

Defined according to Sarnat and Sarnat staging. Moderate to severe birth asphyxia included neonates with Sarnat stage II or III hypoxic-ischemic encephalopathy (HIE).

##### General symptoms

Included lethargy, poor feeding, weak cry, temperature instability (fever > 38 °C or hypothermia < 36.5 °C), apnea, or poor perfusion.

##### Patent ductus arteriosus (PDA)

Diagnosed based on clinical signs (continuous murmur, bounding pulses) and confirmed by echocardiography where available.

##### Shock

Defined as clinical evidence of poor perfusion requiring fluid bolus and/or vasoactive support.

##### Pneumonia

Diagnosed based on respiratory distress with radiological evidence of pulmonary infiltrates and/or consolidation along with clinical suspicion of infection.

##### Meningitis

Defined by positive cerebrospinal fluid (CSF) culture or abnormal CSF parameters with clinical signs suggestive of central nervous system infection.

##### Urinary tract infection (UTI)

Diagnosed by growth of a pathogenic organism in urine culture obtained by sterile catheterization or suprapubic aspiration.

##### Pyoderma

Defined as localized skin infection with purulent lesions and clinical signs of inflammation.

##### Umbilical sepsis

Diagnosed by purulent umbilical discharge with surrounding erythema and systemic signs of infection.

##### Osteomyelitis and septic arthritis

Diagnosed based on clinical features, radiological findings, and/or positive culture from sterile sites.

##### Invasive procedures

Included endotracheal intubation, mechanical ventilation, central venous catheterization, umbilical catheterization, and surgical procedures.

##### Early-onset neonatal infection (EONS)

Infection occurring within the first **72 h of life**.

##### Late-onset neonatal infection (LONS)

Infection occurring **after 72 h of life**.

#### Poor weight gain

Poor weight gain was defined as failure to regain birth weight or excessive weight loss (> 10% of birth weight) within the first 48–72 h, which can be an early indicator of feeding difficulties or underlying illness, including sepsis [5].

#### Definitions of outcome variables

##### Culture-proven bacterial infection

Defined as isolation of a pathogenic organism from blood culture. This group was considered the reference standard for diagnostic accuracy analyses.

##### Septic screen–positive neonates

Neonates with one or more positive parameters in the septic screen as per National Neonatology Forum (NNF) guidelines, irrespective of blood culture results [5]. 

The cut-off values for a positive rapid screening test in this study were as follows:

1. Total leukocyte count < 5000/mm³.
2. Absolute neutrophil count: low counts as per Manroe’s chart for term neonates and Mouzinho’s chart for very low birth weight (VLBW) infants.
3. Immature (band cell) to total neutrophil ratio > 0.2.
4. C-reactive protein (CRP) > 10 mg/L.
5. Micro – ESR (Age of neonates in days + 1).
6. Presence of toxic granules in peripheral smear.

Any one of the above was considered positive sepsis screen.

##### Clinical sepsis

Neonates with clinical signs suggestive of infection but without positive blood culture or septic screen.

#### Group classification

In cases where a neonate fulfilled criteria for more than one category, culture-proven bacterial infection took precedence, followed by septic screen–positive sepsis and then clinical sepsis. Each neonate was assigned to only one group for analysis.

#### Sample size

According to the geographical area, the estimated blood culture positivity rate was 25%–45% [4]. The lower end of the range of (i.e., 25%) was used to optimize the sample size. A minimum sample size of 157 neonates was calculated to detect at least 10% difference around a proportion of 0.25, with a power of 80 and two-sided alpha of 0.05.

### Statistical analysis

Data entry was performed using EpiData Entry 3.1 (Odense, Denmark), and statistical analyses were conducted using Stata version 12 (StataCorp, College Station, TX). Distribution of continuous variables was assessed using visual inspection of histograms and the Shapiro–Wilk test. Normally distributed continuous variables were summarized as mean ± standard deviation and compared using the independent samples t-test. Non-normally distributed variables were summarized as median (interquartile range) and compared using appropriate non-parametric tests. Categorical variables were analyzed using the chi-square test or Fisher’s exact test as appropriate.

Receiver operating characteristic (ROC) curve analysis was performed to evaluate the diagnostic performance of serum ferritin. The optimal cut-off value was determined using the Youden Index (sensitivity + specificity − 1). A p-value < 0.05 was considered statistically significant.

## Results

**Fig. 1:**
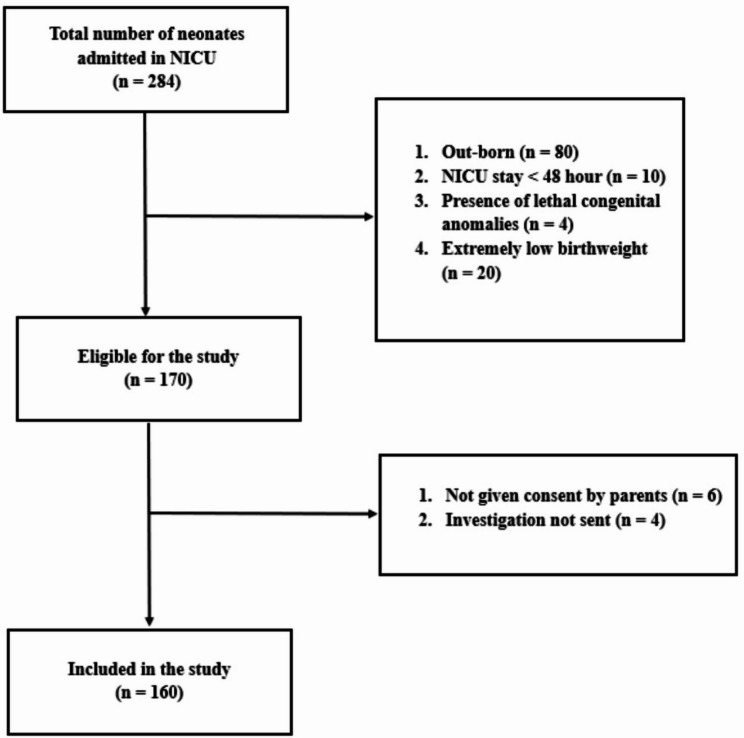
The recruitment process of participants included in the study

In total, 160 neonates were included in the study. Figure 1 shows the recruitment process.

The mean age at admission was 1.83 days (± 2.26 SD). The majority were male patients (*n* = 102, 64%), with the remainder being female patients (*n* = 58, 36%). The mean birth weight of neonates included in the study was 2.37 kg (± 0,58), ranging from 1 to 4 kg. The mean gestational age among term neonates was 37.6 weeks (± 0.51 SD, range 37–40 weeks) and preterm neonates had a mean gestational age of 33.5 weeks (± 1.48 SD, range 31–36). Figure 1 shows the recruitment process of participants included in the study. Table 1 summarizes neonatal and maternal characteristics.

**Table 1:** Neonatal and maternal characteristics of the neonates were included in the study

Neonatal Characteristics	Number (*n* = 160) (%)
Age	
< 1 day	9 (6)
1–5 days	137 (85)
> 5 days	14 (9)
Sex	
Male	102 (64)
Female	58 (36)
Gestational status	
Term	111 (69)
Preterm	49 (31)
Birth weight	
Low birth weight (≤ 2.5 kg)	86 (54)
Normal birth weight (> 2.5 kg)	74 (46)
**Maternal Characteristics**	
Maternal education	
Illiterate	149 (93)
Literate	11 (7)
Previous hospitalization of mother (last 60 days)	
Yes	50 (31)
No	110 (69)
Intake of iron and folic acid during pregnancy	
Yes	88 (55)
No	72 (45)
Gravida	
Primigravida	56 (35)
Multigravida	104 (65)
Mode of delivery	
Vaginal	128 (80)
Caesarian	32 (20)

The time of sending the sepsis screen, blood culture and serum ferritin varied with a median of 37 h, mean ± SD of 57.05 ± 54.59 h and range of 22 to 255 h. The mean ± SD timing of sepsis screening was 58.55 ± 58.56 in neonates with late-onset sepsis and 56.69 ± 60.96 in neonates with early-onset sepsis.

Table 2 presents the Clinical features and risk factors associated with culture-proven bacterial infections.

**Table 2:** Clinical features and risk factors associated with culture-proven bacterial infections

Variable	Total (*n* = 160)	Culture-Positive (*n* = 46)	Culture-Negative (*n* = 114)	OR (95% CI)	*p*-value
General Symptoms					
Signs/Symptoms					
No	67 (42)	39 (85)	28 (25)	R	R
Yes	93 (58)	7 (15)	86 (75)	0.05 (0.02–0.14)	0.001
Lethargy					
No	147 (92)	39 (85)	108 (95)	R	R
Yes	13 (8)	7 (15)	6 (5)	3.23 (1.02–10.20)	0.046
Poor weight gain					
No	145 (91)	37 (80)	108 (95)	R	R
Yes	15 (9)	9 (20)	6 (5)	4.37 (1.45–13.13)	0.008
Refusal to feed					
No	150 (94)	38 (83)	112 (98)	R	R
Yes	10 (6)	8 (17)	2 (2)	11.78 (2.39–57.96)	0.002
Hypothermia					
No	152 (95)	41 (89)	111 (97)	R	R
Yes	8 (5)	5 (11)	3 (3)	4.51 (1.03–19.73)	0.045
Respiratory distress					
No	132 (83)	30 (65)	102 (89)	R	R
Yes	28 (17)	16 (35)	12 (11)	4.53 (1.93–10.62)	0.001
Central nervous system (CNS) signs and symptoms					
Seizures					
No	136 (85)	29 (63)	107 (94)	R	R
Yes	24 (15)	17 (37)	7 (6)	8.96 (3.39–23.66)	0.001
Birth Asphyxia (Stage II/III)					
No	143 (89)	35 (76)	108 (95)	R	R
Yes	17 (11)	11 (24)	6 (5)	5.65 (1.94–16.41)	0.001
Underwent invasive intervention					
No	132 (83)	30 (65)	102 (89)	R	R
Yes	28 (17)	16 (35)	12 (11)	4.53 (1.93–10.62)	0.001
Feeding started in the first 24 h					
No	20 (13)	11 (24)	9 (8)	R	R
Yes	140 (87)	35 (76)	105 (92)	0.27 (0.10–0.71)	0.008

Outcomes included blood culture positivity (28%), septic screen positivity (45%), and clinical sepsis (27%). Ten neonates (6%) died due to sepsis during the study period. Among the 46 culture-positive cases, *Klebsiella pneumoniae* was the most commonly isolated organism (*n* = 20, 43%), followed by *Candida* species (*n* = 11, 25%), *Staphylococcus aureus* (*n* = 7, 16%), *Escherichia coli* (*n* = 4, 8.6%), and *S. hemolyticus* (*n* = 4, 8.3%).

Among the 72 neonates with a positive septic screen, total leukocyte count of < 5000/mm³ was observed in 30% (*n* = 3). Absolute neutrophil count below gestational norms was observed in 11% (*n* = 8); immature (band cell) to total neutrophil (I: T) ratio > 0.2 in 2% (*n* = 3); toxic granules in peripheral smear in 4% (*n* = 3); and positive CRP was observed in 64% (*n* = 46). The mean CRP levels in all neonates and those with culture-positive sepsis (*n* = 46) were 12.01 ± 2.02 (95% CI 8.01–16.00) and 22.90 (95% CI 9.25–36.56), respectively. In neonates with septic screen-positive sepsis (*n* = 72) and those with clinical sepsis (*n* = 44), the mean CRP levels were 18.12 (95% CI 9.35–26.89) and 17.36 (95% CI 5.91–28.8), respectively. Micro-ESR was elevated in 24% (*n* = 39) of all neonates, with an overall mean of 13.86 mm/h (± 1.64).

The mean ferritin levels in all neonates included in the study (*n* = 160) versus those with culture-positive sepsis (*n* = 46) were 317.51 ± 59.2 (95% CI 200.54–438.48) and 326.61 (95% CI 246.55–406.67), respectively.

The mean ferritin levels in neonates with septic screen-positive sepsis (*n* = 72) and those with clinical sepsis (*n* = 44) were 426.9 (95% CI 183.51–670.33) and 283.05 (95% CI 218.12–347.98), respectively.

Serum ferritin alone demonstrated superior diagnostic accuracy in predicting culture-positive sepsis, with an area under the ROC curve (AUC) of 0.70 compared to sepsis screen alone (AUC 0.58) (Table 3).

**Table 3:** Receiver operating characteristic (ROC) curve analysis of septic screen, serum ferritin and combined serum ferritin (> 295.5 ng/mL) with septic screen using the Youden index against blood culture as reference standard

Reference Outcome-Blood culture positive sepsis	AUC	Youden Cut-Off (ng/mL)	Youden Index (J)	Sensitivity	Specificity	Positive predictive value	Negative predictive value
Sepsis screen	0.58	-	-	57%	60%	36%	77%
Serum Ferritin	0.70	295.5	0.403	54%	86%	-	99%
Serum Ferritin included in sepsis screen	0.59	295.5	0.182	72%	46%	35%	80%

At the optimal cut-off value of 295.5 ng/mL (based on the Youden Index), it achieved a sensitivity of 54% and specificity of 86% (Youden Index: 0.403). In comparison, the septic screen alone showed an AUC of 0.58, indicating limited diagnostic power. When serum ferritin (> 295.5 ng/mL) was combined with a positive septic screen, sensitivity increased to 72%, but specificity decreased to 46%, with a lower Youden Index of 0.182, reflecting poorer overall discriminatory power. Serum ferritin exhibited a strong negative predictive value of 99% for ruling out sepsis.

## Discussion

In our study, invasive interventions were significantly associated with culture-positive sepsis beyond birth weight and gestational age. A multicentric neonatal sepsis study across 12 resource-limited sites reported risk factors related to healthcare exposure, maternal history, birth conditions, and living environment [15]. Birth asphyxia was a significant predictor (relative risk: 3·82), closely matching the OR of 5.65 in our study (95% CI 1.94–16.41; *p* = 0.001) [15].

We observed a 28% blood culture positivity rate among neonates suspected of sepsis in a Level-III NICU. This rate aligns with or exceeds culture-confirmed sepsis incidence in broader populations, especially in resource-limited settings. Global estimates suggest clinical neonatal sepsis ranges from 49 to 170 cases per 1,000 live births in LMICs, while blood culture-confirmed sepsis is much lower, around 5.5 per 1,000 live births [3, 8]. India has a high estimated clinical neonatal sepsis incidence of 17,000 per 100,000 live births [4]. Our findings reflect concentrated cases in tertiary care NICU setting and local diagnostic challenges.

Regarding pathogen spectrum, *Klebsiella pneumoniae* was the most commonly isolated organism (43%, *n* = 20) in our cohort, followed by *Candida* species [15]. This Gram-negative prevalence aligns with LMIC findings where these pathogens frequently cause neonatal sepsis. The significant *Candida* presence is noteworthy, as it is not typically among leading causes. Its frequent isolation likely reflects true bloodstream infections attributable to broad-spectrum antibiotic use, prolonged invasive procedures, partial parenteral nutrition, and immature neonatal immunity [6, 7]. These factors collectively foster fungal colonization, contributing to increased Candidiasis incidence in our cohort. High mortality (10/46 culture-positive cases) reflects severe disease, multi-drug-resistant pathogens like *Klebsiella pneumoniae*, and comorbidities including prematurity (31%, *n* = 49) and low birth weight (54%, *n* = 86) [6, 7].

Consistent with prior research, our study identified significant risk factors for culture-positive neonatal sepsis. Clinical symptoms including lethargy, poor weight gain, refusal to feed, hypothermia, respiratory distress, and seizures were significantly linked to culture-confirmed sepsis [Table 2], aligning with the documented non-specific clinical presentation posing diagnostic challenges [3, 5–7]. These signs reflect the systemic nature of infection in neonates whose immature immune systems respond less overtly [5].

Invasive interventions were significantly associated with culture-positive sepsis [Table 2], consistent with literature in tertiary care settings. Procedures including central venous catheterization and mechanical ventilation are recognized portals for pathogen entry, increasing healthcare-associated infection risk [4, 15].

Birth asphyxia emerged as a significant predictor of culture-positive sepsis [Table 2], aligning with multicentric studies highlighting birth conditions as key risk factors [15]. Physiological stress from birth asphyxia compromises neonatal immune function and disrupts gut barrier integrity, increasing bacterial translocation susceptibility [15]. The observed odds ratio of 5.65 closely mirrors broader cohort findings.

Risk factor prevalence and contribution vary by geography, socioeconomic conditions, healthcare resources, and infection control practices. The high neonatal sepsis burden in India underscores regional context [4]. Frequent invasive procedures and challenges in preventing perinatal asphyxia in resource-constrained settings contribute to observed associations.

We observed protective effects of early feeding initiation on culture-confirmed sepsis [Table 2]. Early enteral nutrition in preterm infants positively impacts outcomes, with exclusive enteral feeding associated with lower late-onset sepsis incidence [16]. Conversely, delayed enteral feeding increases sepsis hazard due to impaired gut motility and heightened gut-related complication susceptibility [16]. This protection stems from establishing robust gut microbiome and improved gut barrier function, crucial for neonatal immune system development [16].

CRP remains the most studied biomarker for neonatal sepsis; however, its sensitivity is reduced in early-onset sepsis as serum levels peak 36–48 h after infection onset [3, 6, 7, 12, 15–17]. CRP levels may increase in non-infectious maternal and neonatal conditions, limiting specificity, though it offers high negative predictive value for guiding antibiotic discontinuation when measured serially [3, 6, 12].

A critical aspect involves understanding confounding factors affecting sepsis screen components and ferritin. CRP, a key sepsis screen component, can increase in various non-infectious conditions, potentially causing false-positive results and unnecessary antibiotic exposure [3, 6, 12]. Other inflammatory markers can be elevated in perinatal asphyxia, meconium aspiration syndrome, and intracranial hemorrhage, confounding true infection diagnosis [19].

Serum ferritin, an acute-phase reactant, is elevated in bacterial sepsis, viral infections, and non-sepsis conditions including perinatal asphyxia, potentially leading to false positives in neonates prone to inflammatory states [13, 19, 20]. Studies show increased ferritin in preterm premature rupture of membranes due to infection-induced acute phase response [21]. Gestational age and birth weight independently influence baseline ferritin levels, potentially causing false negatives with universal cut-offs [22].

Our study inclusion of neonates with hypoxic-ischemic encephalopathy enhances external validity, reflecting real-world NICU scenarios where sepsis coexists with severe conditions [15]. Birth asphyxia remains a significant sepsis risk factor (OR 5.65, *p* = 0.001) [Table 2]. The generalizability of the ferritin cut-off (> 295.5 ng/mL) requires consideration as baseline levels vary with gestational age, postnatal age, birth weight, and ethnicity, necessitating future research for adjusted reference ranges.

The conventional sepsis screen demonstrated limited diagnostic power (AUC 0.58, sensitivity 57%, specificity 60%) [Table 3], consistent with literature concerns about variable traditional screen components [22–24]. Serum ferritin alone showed superior diagnostic accuracy (AUC 0.70, sensitivity 54%, specificity 86%) at > 295.5 ng/mL [Table 3], with high negative predictive value (99%) for ruling out sepsis. While combining ferritin with the sepsis screen increased sensitivity to 72%, specificity decreased to 46% (Youden Index 0.182) [Table 3], introducing more false positives due to ferritin elevation in non-sepsis inflammatory conditions [19, 20]. This highlights limited role of serum ferritin in neonatal sepsis diagnosis despite enhanced early detection potential in resource-limited settings.

While our study was conducted in a cohort of neonates with suspected sepsis where the prevalence of culture-positive sepsis was 28%, applying this 99% NPV directly to a general neonatal population, which has a much lower baseline incidence of culture-proven sepsis [3, 8,] would require careful consideration. The generalizability of such a high NPV, therefore, necessitates further validation across diverse populations with varying disease prevalence rates [20], acknowledging that factors like gestational age, postnatal age, and ethnic differences can influence biomarker expression and diagnostic performance [22]. Thus, while highly encouraging, the utility of this 99% NPV in differing clinical contexts should be carefully evaluated to avoid overestimation and ensure appropriate clinical decision-making.

The systemic inflammatory response in neonatal sepsis triggers iron homeostasis alterations and ferritin elevation, linked to pathophysiological changes during infection that activate the autonomic nervous system [25, 26]. This ferritin increase involves interplay between inflammation, iron metabolism, and oxidative stress, where ferritin sequesters iron [22].

Neonates exhibit physiologically elevated fetal hemoglobin, ferritin, and serum iron, with immune system immaturity complicating diagnostics as inflammatory markers may not show robust responses [27, 28]. Gestational age and birth weight influence baseline ferritin levels and infection response magnitude [22]. Consequently, establishing stratified ferritin reference ranges is crucial for accurate diagnostic interpretation, particularly given varied physiological iron stores across diverse populations requiring universally applicable normative values [23, 24, 28].

This study highlights serum ferritin’s limited diagnostic utility for neonatal sepsis (AUC 0.70) [29, 30], though surpassing conventional sepsis screen (AUC 0.58). A 295.5 ng/mL cutoff demonstrated balanced sensitivity (54%) and specificity (86%) with high negative predictive value (99%), potentially reducing unnecessary antibiotic exposure. This is crucial as blood culture, the gold standard, often yields false negatives or delays intervention [31]. Blood culture delays and sample limitations necessitate alternative biomarkers [3, 29, 32].

Low blood culture yield leads NICUs to rely on composite sepsis screens (total leukocyte count, absolute neutrophil count, immature-to-mature neutrophil ratio, micro-ESR, CRP) [5]. However, conventional screens demonstrate limited sensitivity and specificity, contributing to increased empirical antibiotic use, disrupted gut microbiota, and multidrug-resistant organisms [8–11]. Improving accuracy is urgently needed [3, 6, 8, 12].

Serum ferritin demonstrated superior diagnostic accuracy (AUC 0.70, sensitivity 54%, specificity 86% at > 295.5 ng/mL), with strong negative predictive value (99%). Incorporating ferritin increased sensitivity to 72% but decreased specificity to 46% (Youden Index 0.182), indicating poorer discriminatory power.

Previous research supports ferritin’s utility. A Romanian study reported significantly higher ferritin in neonates with culture-confirmed sepsis [3, 29]. A pediatric ICU study found median peak ferritin of 150.5 ng/mL in patients with sepsis, with 10-fold increase associated with 5-fold mortality risk. Ferritin’s AUC for mortality prediction was 0.787 [33].

Serum ferritin, an acute-phase protein, can be elevated in various conditions beyond bacterial sepsis, including systemic lupus erythematosus, rheumatoid arthritis, cancers, cardiovascular conditions, viral infections, tuberculosis, and malaria [13]. In neonates, elevated ferritin occurs in non-sepsis conditions like perinatal asphyxia [19, 20]. A Kerala study found higher ferritin in pregnant women with preterm premature rupture of membranes [21].

Viral infections can trigger complex systemic effects, including immune dysregulation, organ damage, endotoxin-mediated inflammatory responses, and severe complications such as long COVID manifestations and fulminant hepatic failure during pregnancy [34–36].

Ferritin elevation during sepsis involves acute phase response and reticuloendothelial system activation [20]. Gestational age and birth weight independently influence baseline ferritin levels and infection response, necessitating age- and weight-adjusted reference ranges [22]. Future investigations should examine ferritin isoforms for nuanced pathogenesis insights [37]. Varied physiological iron stores across populations necessitate further research for universally applicable normative ferritin values [23, 24].

Ferritin’s high negative predictive value suggests potential for safe antibiotic de-escalation in neonates with suspected sepsis but low ferritin levels, reducing unnecessary antibiotic exposure risks like antimicrobial resistance and microbiome dysbiosis. Integrating ferritin into diagnostic algorithms could facilitate nuanced risk stratification for tailored management strategies optimizing patient care and antibiotic stewardship [38, 39]. However, generalizability requires validation in diverse pediatric cohorts given single-center limitations in biomarker research [20].

The sepsis screen’s lower AUC (0.58) indicates limited standalone utility, as markers like CRP may elevate in non-infectious conditions including perinatal asphyxia, meconium aspiration syndrome, and intracranial hemorrhage, confounding infection diagnosis [19, 38]. A Kerala study found significantly higher serum ferritin levels in PROM patients versus controls [21].

A pediatric ICU study of 312 patients with anemia and sepsis reported median peak ferritin of 150.5 ng/mL, significantly associated with mortality (*p* < 0.001), with 10-fold ferritin increase correlating with 5-fold mortality risk and AUC 0.787 for mortality prediction [33]. A Romanian study of 86 neonates (51 culture-confirmed sepsis) reported significantly higher ferritin levels versus non-sepsis group, concluding serum ferritin is a reliable predictive biomarker [40].

### Appropriate timing of investigations

Timing of sepsis screen components and serum ferritin measurements is crucial for diagnostic accuracy. CRP, a common sepsis screen component, typically peaks 36–48 h after infection onset [3, 6, 12]. This delayed response limits sensitivity for early-onset sepsis, and single early CRP measurements may yield false negatives, necessitating serial measurements to monitor trends for guiding antibiotic discontinuation [3, 6, 12].

Serum ferritin levels undergo dynamic changes during sepsis. Our study relied on single time-point measurement, potentially overlooking diagnostic/prognostic potential by not capturing kinetic profiles. Physiological changes in ferritin concentrations due to gestational age, postnatal age, and iron stores complicate single-point measurement interpretation [22–24]. Establishing optimal timing for ferritin measurement, including longitudinal assessments, is critical. Serial measurements could enhance diagnostic and prognostic utility of traditional sepsis markers and novel biomarkers. The absence of data on optimal serial measurement timing for ferritin is a limitation, and future research incorporating longitudinal ferritin measurements is warranted to establish kinetic profiles enhancing diagnostic accuracy and providing prognostic indicators for disease severity and treatment response.

### Strengths of the study

The study’s findings indicate that serum ferritin, with an AUC of 0.70, demonstrates superior diagnostic performance for neonatal sepsis compared to the conventional sepsis screen alone. Its notable 99% negative predictive value suggests a potential role in safely ruling out sepsis, thereby informing clinical decisions to de-escalate or avoid unnecessary empirical antibiotic exposure in NICU practice. This is particularly relevant for guiding antibiotic stewardship in resource-limited settings.

### Limitations

Despite these promising findings, several limitations warrant consideration, including the relatively small sample size and the single-centre nature of the study, which may limit the generalizability of the results to broader populations of neonates [29, 41]. Furthermore, the lack of a standardized ferritin assay across different laboratories could introduce variability in measurements, impacting the comparability and reproducibility of the findings. Additionally, the study’s reliance on a single time point for ferritin measurement may overlook dynamic changes in its levels that could offer further diagnostic or prognostic insights, especially given the rapid progression of neonatal sepsis. Future studies should therefore incorporate longitudinal ferritin measurements, alongside other inflammatory markers, to establish kinetic profiles that could enhance diagnostic accuracy and provide prognostic indicators for disease severity and treatment response [42].

## Conclusions

Mean serum ferritin levels were higher in septic screen-positive neonates than in culture-positive sepsis. Incorporating serum ferritin (cut-off > 295 ng/mL) into the septic screen improved its sensitivity and serum ferritin might have a limited role in diagnosis of neonatal sepsis in culture-confirmed cases and in resource-constrained settings.

## Supplementary Information

Below is the link to the electronic supplementary material.

Supplementary Material 1

## Data Availability

The dataset used and/or analysed during the current study is available from the corresponding author on reasonable request. Individual data can due to confidentiality reasons not be made public. All enquiries regarding data sharing should be made to- The Chairman, Institutional Ethics Committee, R D Gardi Medical College, Agar Road, Ujjain, India 456006. The name of data set corresponding to the study is Diagnostic Utility of Serum Ferritin in Neonatal Sepsis data.

## Abbreviations

CBC: Complete Blood Count
CI: Confidence Interval
CRP: C-reactive Protein
LMICs: Low- and Middle-Income Countries
NICU: Neonatal Intensive Care Unit
NNF: National Neonatology Forum
RBS: Random Blood Sugar
ROC: Receiver Operating Characteristic
SD: Standard Deviation
VLBW: Very Low Birth Weight
ESR: Erythrocyte Sedimentation Rate
