# Supplementary material for: Diagnostic utility of serum ferritin in culture proven bacterial infection: an observational study
Source: BMC Infect Dis. 2026 Apr 15;26:770. doi: 10.1186/s12879-026-13298-3 (PMC13085344; doi:10.1186/s12879-026-13298-3)
Supplement: Supplementary file 1 — Supplementary Material 1 [file 12879_2026_13298_MOESM1_ESM.pdf]

Diagnostic Utility of Serum Ferritin in Neonatal Sepsis: An Observational Study

Questionnaire

Demographic information

IPD No.____________________Name of baby _____________Age at the time of admission________Sex ☐M /☐ F DOB - _________________Time of birth - ___________PM / AM Birth weight_____________(In Kg)

Weight on admission ________(In Kg) Date of admission __________Religion: _______

Date of discharge __________________________________Date of death______________

Mother Information

Blood group baby/mother ____________________Mother's name___________________

Term☐/ preterm ☐SGA☐ AGA☐LGA☐

Gestational age ___________(by new ballard's score)

Referral from facilities

☐Home delivery ☐PHC ☐District hospital ☐Private OPD

☐Private nursing home ☐Self ☐If out born

☐Previous hospitalization – ☐Yes ☐No

Transport time for transfer to CRGH________________________

Antenatal Data

1. Iron and folic acid intake: ☐Yes ☐No
2. Maternal delivery data:
   A. Place of delivery:
      ☐Home: Untrained ☐Trained (☐Dai ☐Nurse☐None)
      ☐Institution: ☐PHC☐District Hospital ☐Private nursing home
   B. Mode of delivery: ☐Vaginal☐Caesarian ☐Assisted

Maternal risk factor's

1. Maternal education:____________________
2. Parental education:____________________
3. Gestation history:

Gravida _______Para ______Abortion ________Living ________Still births_______Death ________

Neonatal Risk Factors

a. **Feeding - ☐Yes ☐No**

   A. Feeding method:

      RTF - ☐Yes ☐No Katori spoon - ☐Yes ☐No DBF - ☐Yes ☐No

   B. Prelacteal feed given: ☐Yes ☐No

   C. Home remedies tried: ☐Yes ☐No

b. **Co-morbidities**

- Respiratory distress syndrome (maximum respiratory score)____________________
- Patent ductus arteriosus ______________________________
- Intraventricular hemorrhage__________________

c. **Birth asphyxia- ☐Yes ☐No**

   If Yes – HIE Stage – I / II / III

d. Umbilical catheter (central venous catheter) inserted – ☐Yes ☐No

e. Exchange transfusion - ☐Yes ☐No

f. endotracheal intubation – ☐Yes ☐No

g. Ventilation - ☐Yes ☐No

h. **Mode of delivery** – Vaginal / LSCS / Assisted vaginal

   ☐ if assisted vaginal- vaccum application ☐outlet forceps

Clinical examination at admission

1) Superficial infection :

Umblical sepsis: ☐Yes ☐No

Pyoderma: ☐Yes ☐No

Conjunctivitis: ☐Yes ☐No

Apneic spell: ☐Yes ☐No

2) **Symptomatology**

Lethargy – ☐Yes ☐No

Refusal to feed – ☐Yes ☐No

Poor cry – ☐Yes ☐ No

Incessant cry – ☐Yes ☐No

Poor weight gain – ☐Yes ☐ No

3) Respiratory examination-

Respiratory rate________min

Chest retraction___________

Grunt ______________

Apnea __________________

4) Central nervous system examination:

Seizure – ☐Yes ☐ No

Bulging anerior frontanalle – ☐Yes ☐No Poor neonatal reflex – ☐Yes ☐ No

5) Gastrointestinal examination:

Abdominal distension ☐Yes ☐ No

Vomiting ☐Yes ☐No

Diarrhoea ☐Yes ☐ No

6) Shock:

Fever ☐Yes ☐No

Hypothermia ☐Yes ☐No

7) Others:

Bleeding ☐Yes ☐No

Sclerema ☐Yes ☐No

8) Diagnosis categories for newborn with sepsis

- Clinical or suspected sepsis, septic screen negative __________________
- Septic screen positive, culture negative sepsis ______________________
- Confirmed sepsis- early onset sepsis ________________late onset sepsis _______________

9) Site of sepsis:

Pneumonia ( ) meningitis ( ) Blood stream infection ( ) UTI ( ) Pyoderm ( )
Umbilical sepsis ( ) Osteomylitis ( ) Septic arthritis ( )

10) Outcome:

1. Culture positive sepsis: (Defined as the presence of bacteria (culture positive) in sterile body fluids like blood, urine, CSF, pleural fluid, or peritoneal fluid. Neonatal sepsis includes septicemia, pneumonia, meningitis, osteomyelitis, arthritis, and urinary tract infection)
2. Positive septic screen: (The cut-off values for a positive rapid screening test in this study were as follows:
   - Total leukocyte count <5000/mm^3
   - Absolute neutrophil count: low counts as per Manroe's chart for term neonates and Mouzinho's chart for very low birth weight (VLBW) infants
   - Immature (band cell) to total neutrophil ratio >0.2
   - C-reactive protein (CRP) >10 mg/L
   - Micro – ESR (Age of neonates in days +1)
   - Presence of toxic granules in peripheral smear

Any one of the above was considered positive sepsis screen.

3. Clinical suspected sepsis (Clinical sepsis was defined according to NNF guidelines as the presence of clinical signs or symptoms with no other recognized cause: tachycardia (heart rate >160/min), tachypnea (RR >60/min) and temperature instability (fever >100.4 F) or hypothermia (<36.5 or 97.7F)

Laboratory test:

(Annexure-2)

Name of Neonate		Age:	Sex:	Date:	
Department		Unit:	OPD No:	Unique Id:	
**Total leucocyte count**					
Neutrophil		Lymphocyte		Basophils	
Eosinophil		MCH		MCV	
MCHC		Absolute neutrophil count		Micro ESR	
**Immatute to mature neutrophils**					
Band cells		Yes / No	Toxic Granules	Yes / No	
Platelet		Peripheral smear		Blood group mother and baby	
**Lumbar puncture**					
Cells		Ps comment		Glucose	
Protein		CSF c/s:			
Blood c/s		S.CRP		S. Ferritin	
**Electrolytes**					
Serum Sodium		Serum K		serum Ca	
**RFT**					
Serum			Serum Creatine		
**LFT**					
NBIL			Albumin		
BU			BC		

Antibiotic use data

Date of start of antibiotics______________

Name of antibiotics_________Dose (in mg)____Frequency ____Route ______Duration in day______
